# Supplementary material for: High-Throughput Microfluidic Production of Ultrasmall Lecithin Nanoliposomes for High-Efficacy Transdermal Delivery and Skin-Aging Treatment
Source: Biomedicines. 2025 Jan 30;13(2):322. doi: 10.3390/biomedicines13020322 (PMC11853437; doi:10.3390/biomedicines13020322)
Supplement: Supplementary file 1 [file biomedicines-13-00322-s001.zip › biomedicines-3424777-supplementary.pdf]

Supporting Information

High-throughput microfluidic production of ultrasmall lecithin nanoliposomes for high-efficacy transdermal delivery and skin-aging treatment

*Xiao Liang[1, 2], Chan Lu[1, 2], Fangqiao Zheng[1, 2], Zhengyi Lan[2], Haoji Wang[1, 2], Muhammad Shafiq[3], Xinxin Pan[4, *], Hangrong Chen[1, 2] and Ming Ma[1,2, *]*

[1] School of Chemistry and Materials Science, Hangzhou Institute for Advanced Study, University of Chinese Academy of Sciences, Hangzhou 310024, P. R. China.

[2] State Key Laboratory of High Performance Ceramics and Superfine Microstructures, Shanghai Institute of Ceramics, Chinese Academy of Sciences, Shanghai 200050, P. R. China.

[3] Innovation Center of NanoMedicine (iCONM), Kawasaki Institute of Industrial Promotion, Kawasaki-ku, Kawasaki 210-0821, Japan.

[4] School of Biotechnology, East China University of Science and Technology, Shanghai 200237, P. R. China.

[*] Correspondence: Xinxin Pan: y10220060@mail.ecust.edu.cn, Ming Ma: mma@mail.sic.ac.cn.

Table of Contents

Skin Patch Test

Supplementary Figures and Tables

Figure S1. Image of a printed HBSCF device used for nanoliposomes synthesis.

Figure S2. DLS particle size and PDI values of the nanoliposomes obtained at different TFR and a fixed FRR value of 40.

Figure S3. Normalized size distribution plots of nanoliposomes synthesized with an HBSCF device and a CF device and the at a fixed TFR value of 80 mL/min and FRR value of 40.

Figure S4. Cryo-TEM images of NLP_{NM} synthesized with an HBSCF device and a CF device at a fixed TFR value of 10 mL/min and FRR value of 40.

Figure S5. Normalized size distribution plots of CoQ10 and DiI loaded NLP_{US} prepared with an HBSCF device at a fixed TFR value of 80 mL/min and FRR value of 40.

Figure S6. DLS particle size and PDI values of the nanoliposomes loaded with CoQ10 obtained at different TFR and a fixed FRR value of 40.

Figure S7. Variation in particle size of the CoQ10@NLP_{US} prepared with an HBSCF device over 48 hours.

Figure S8. Confocal images showing cellular uptake of DiI and DiI@NLP_{US} at different time points.

Figure S9. Confocal images showing the cellular uptake of DiI and DiI@NLP_{US} at 10 min.

Figure S10. In vitro cytotoxicity of the free CoQ10, blank NLP_{US} and CoQ10@NLP_{US} against HSF cells detected by CCK-8.

Figure S11. The cellular ROS levels detected by flow cytometry.

Figure S12. Body weights of ICR mice treated with PBS, CoQ10, NLP_{US} or CoQ10@NLP_{US}.

Figure S13. Routine blood analysis.

Figure S14. Blood biochemical analysis.

Figure S15. Histological analysis of major organs from mice treated with PBS, CoQ10, NLP_{US}, and CoQ10@NLP_{US} at 2 weeks post-operatively.

Table S1. Multiple skin irritation test of PBS, CoQ10, $NLP_{US,}$ and CoQ10@NLP_{US} on 20 ICR mice.

Skin Patch Test

Skin irritation test of CoQ10@NLP_{US} was performed according to Safety and Technical Standards for Cosmetics 2015. Twenty normal-grade female ICR mice were shaved on the dorsal spine while avoiding the damage of the epidermis. The area of hair removal was approximately 2 cm × 2 cm. About 300 μL of drug (PBS, CoQ10, NLP_{US}, CoQ10@NLP_{US}) with Aquaphor (Eucerin, Germany) was applied to the skin.

Skin was shaved before each application from the second day onwards, and the residue was removed with warm water, and the local reaction was observed and scored after 1 h. The Erythema scoring criterion were as follows: criteria: 0, no erythema; 1, slight erythema; 3, apparent erythema; 4, purplish red erythema and scorch. Edema scoring criteria: 0, no edema; 1, slight edema; 2, skin elevation with precise contours; 3, skin elevation about 1 mm; 4, skin elevation 1 mm. The mean score per animal daily was calculated to assess the skin irritation intensity: 0 to <0.5, no irritation; 0.5 to 2.0, light irritation; 2.0 to 6.0, moderate irritation: 6.0-8.0, intense irritation. It can be concluded from Table S1 that CoQ10, NLP_{US} and CoQ10@NLP_{US} had no irritation.

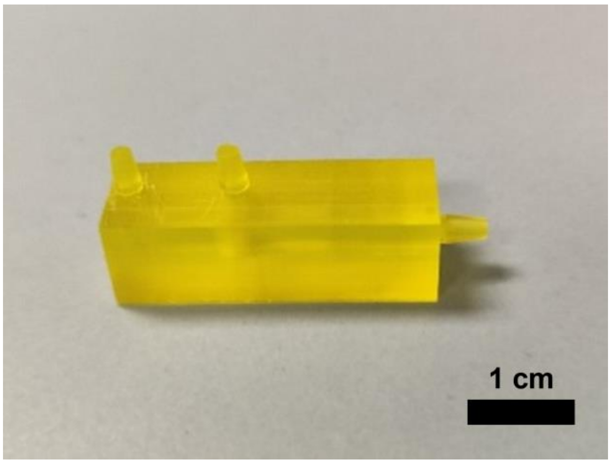

Figure S1. Image of a printed HBSCF device used for nanoliposomes synthesis (Scale bar: 1 cm).

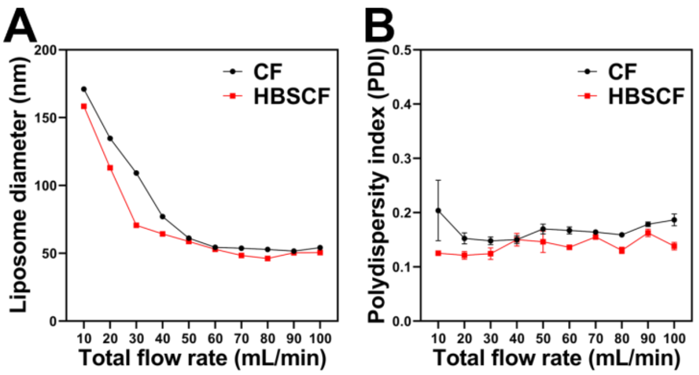

Figure S2. DLS particle size (A) and PDI values (B) of the nanoliposomes obtained at different TFR and a fixed FRR value of 40. The values are represented as the mean ± SEM.

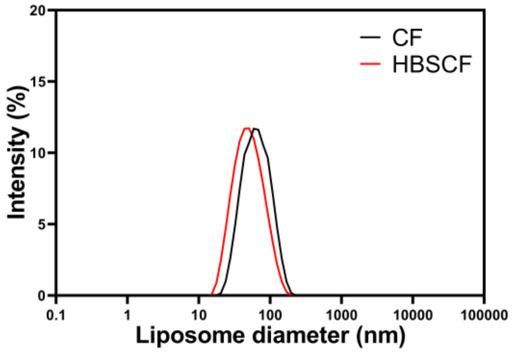

Figure S3. Normalized size distribution plots of nanoliposomes synthesized with an HBSCF device and a CF device and the at a fixed TFR value of 80 mL/min and FRR value of 40.

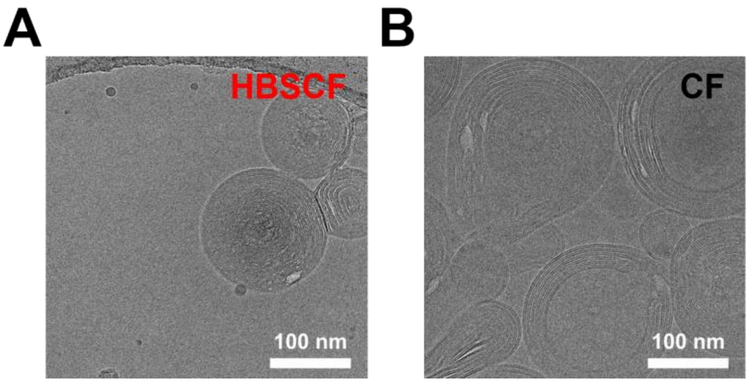

Figure S4. Cryo-TEM images of NLP_{NM} synthesized with an HBSCF device (A) and a CF device (B) at a fixed TFR value of 10 mL/min and FRR value of 40 (Scale bar: 100 nm).

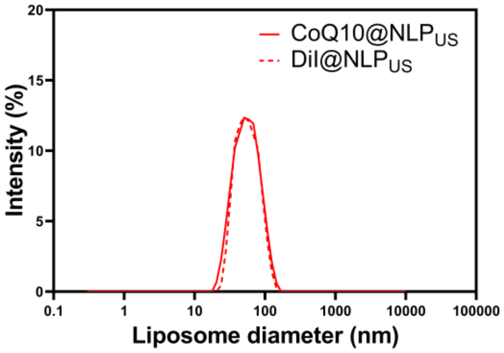

Figure S5. Normalized size distribution plots of CoQ10 and DiI loaded NLP_{US} prepared with an HBSCF device at a fixed TFR value of 80 mL/min and FRR value of 40.

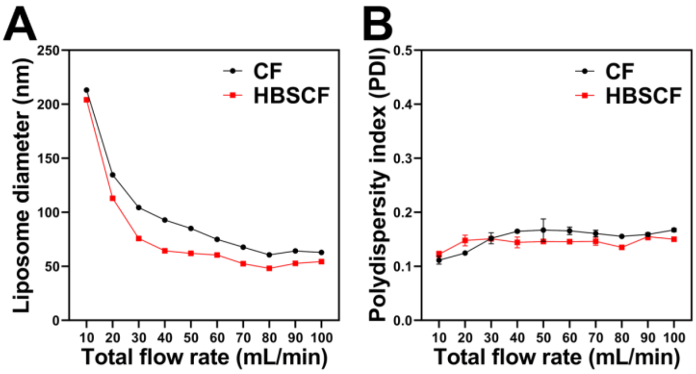

Figure S6. DLS particle size (A) and PDI values (B) of the nanoliposomes loaded with CoQ10 obtained at different TFR and a fixed FRR value of 40. The values are represented as the mean ± SEM.

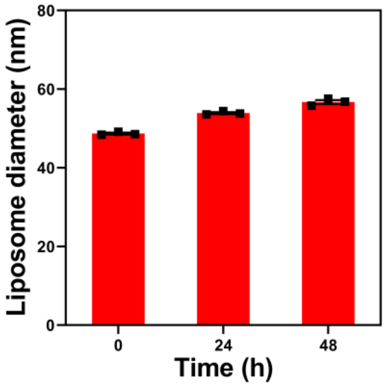

Figure S7. Variation in particle size of the CoQ10@NLP_{US} prepared with an HBSCF device over 48 hours. The values are represented as the mean ± SEM.

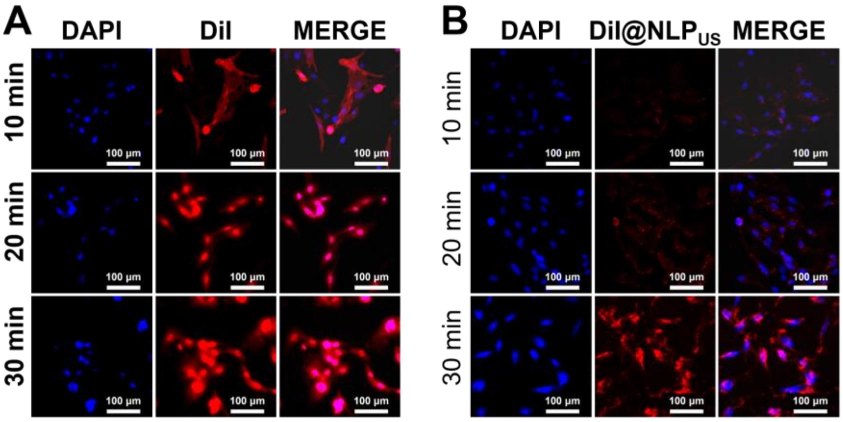

Figure S8. Confocal images showing cellular uptake of DiI (A) and DiI@NLP$_{US}$ (B) at different time points (Scale bar: 100 μm).

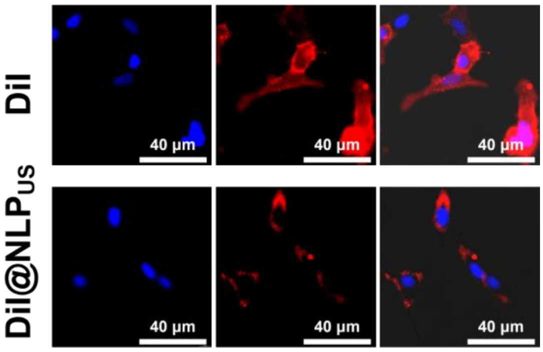

Figure S9. Confocal images showing the cellular uptake of DiI (A) and DiI@NLP$_{US}$ (B) at 10 min (Scale bar: 40 μm).

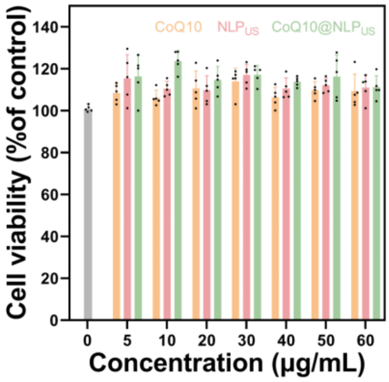

Figure S10. In vitro cytotoxicity of the free CoQ10, blank NLP$_{US}$ and CoQ10@NLP$_{US}$ against HSF cells detected by CCK-8. Cells were treated with free CoQ10, blank NLP$_{US}$ and CoQ10@NLP$_{US}$ at the same CoQ10 or lipid concentrations over a wide range (0-60 μg/mL). Cells treated with DMEM only served as the control group. The values are represented as the mean ± SEM.

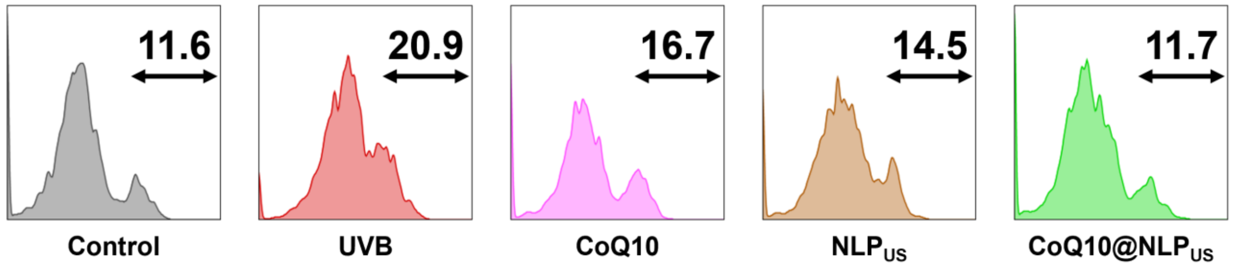

Figure S11. The cellular ROS levels detected by flow cytometry.

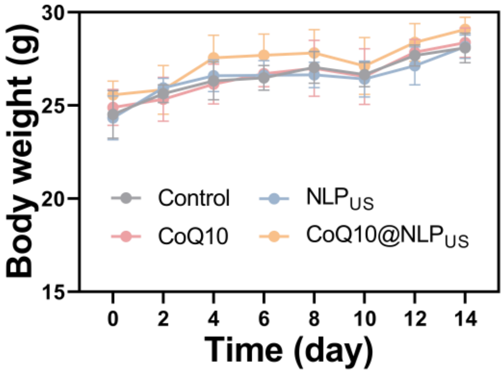

Figure S12. Body weights of ICR mice treated with PBS, CoQ10, NLP_{US} or CoQ10@NLP_{US}. The values are represented as the mean ± SEM.

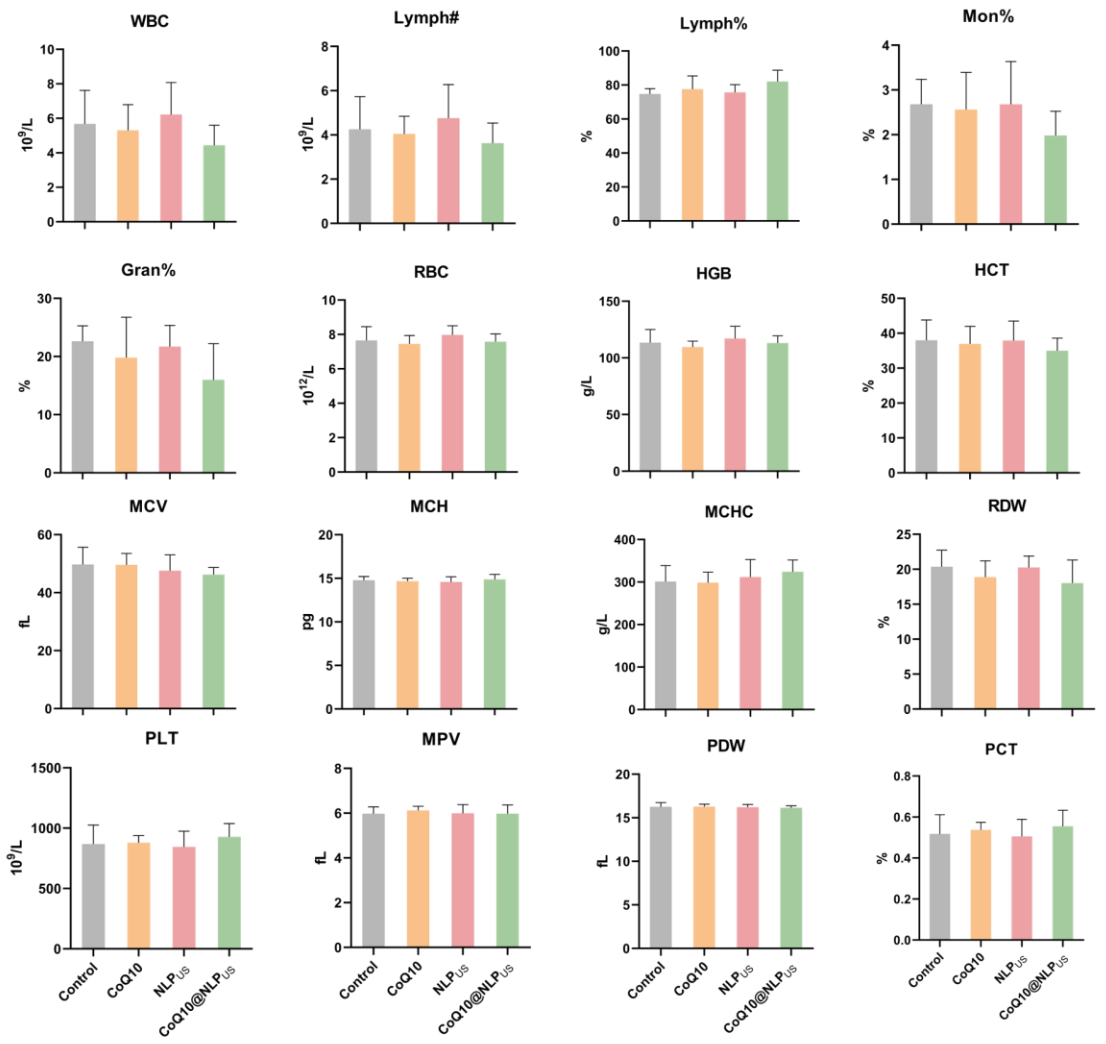

Figure S13. Routine blood analysis. White blood cell count (WBC), lymphocyte count (Lymph#), lymphocyte ratio (Lymph%), monocyte ratio (Mon%), granulocytes ratio (Gran%), red blood cell count (RBC), hemoglobin (HGB), hematocrit (HCT), mean corpuscular volume (MCV), mean corpuscular hemoglobin (MCH), mean corpuscular hemoglobin concentration (MCHC), red cell distribution width (RDW), platelet (PLT), mean platelet volume (MPV), platelet distribution width (PDW), and thrombocytocrit (PCT) levels in the blood samples of PBS, CoQ10, NLP$_{US}$, and CoQ10@NLP$_{US}$ group. All behavioral test bar or plot charts are presented as the mean ± SEM.

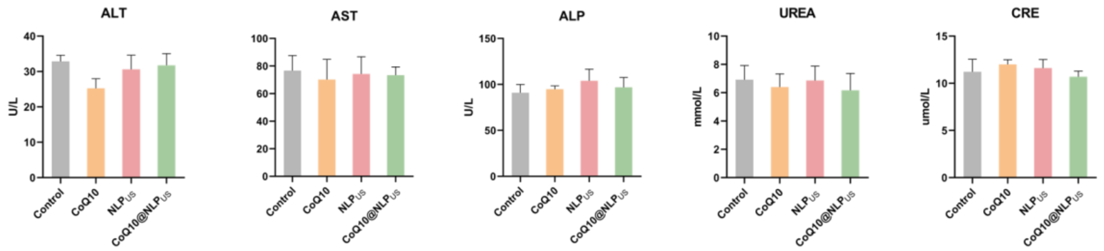

Figure S14. Blood biochemical analysis. Alanine aminotransferase (ALT), aspartate aminotransferase (AST), serum alkaline phosphatase (ALP), urea (UREA), and creatinine (CREA) levels in the blood samples of all group. All behavioral test bar or plot charts are presented as the mean ± SEM.

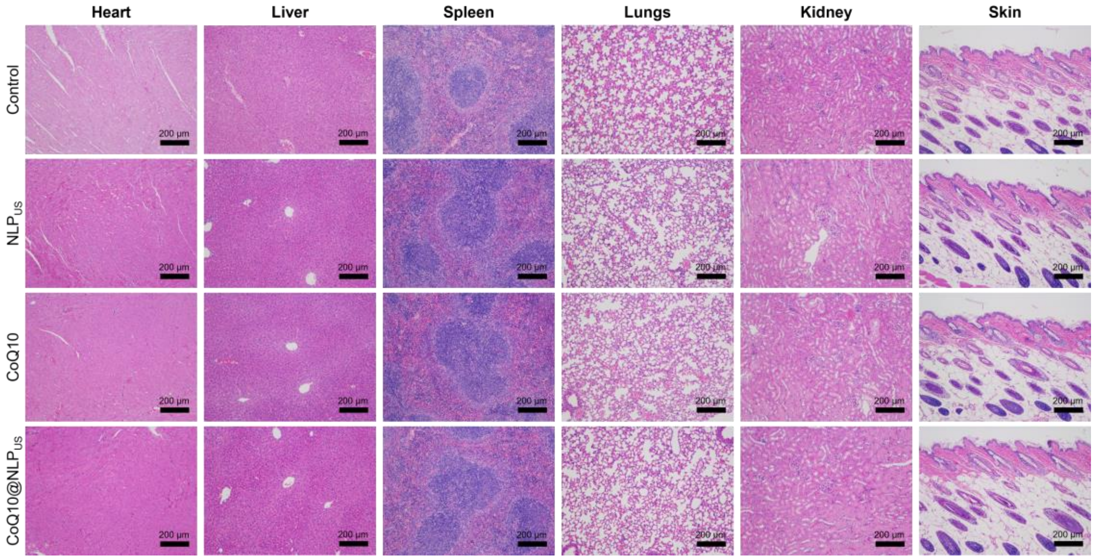

Figure S15. Histological analysis of major organs from mice treated with PBS, CoQ10, NLP_{US}, and CoQ10@NLP_{US} at 2 weeks post-operatively (Scale bar: 200 μm).

Table S1. Multiple skin irritation test of PBS, CoQ10, NLP_{US}, and CoQ10@NLP_{US} on 20 ICR mice.

Days	Animal population	Stimulus response score											
		CoQ10			NLP_{US}			CoQ10@NLP_{US}			Control		
		Erythema	Edema	Sum	Erythema	Edema	Sum	Erythema	Edema	Sum	Erythema	Edema	Sum
1	20	0	0	0	0	0	0	0	0	0	0	0	0
2	20	0	0	0	0	0	0	0	0	0	0	0	0
3	20	0	0	0	0	0	0	0	0	0	0	0	0
4	20	0	0	0	0	0	0	0	0	0	0	0	0
5	20	0	0	0	0	0	0	0	0	0	0	0	0
6	20	0	0	0	0	0	0	0	0	0	0	0	0
7	20	0	0	0	0	0	0	0	0	0	0	0	0
8	20	0	0	0	0	0	0	0	0	0	0	0	0
9	20	0	0	0	0	0	0	0	0	0	0	0	0
10	20	0	0	0	0	0	0	0	0	0	0	0	0
11	20	0	0	0	0	0	0	0	0	0	0	0	0
12	20	0	0	0	0	0	0	0	0	0	0	0	0
13	20	0	0	0	0	0	0	0	0	0	0	0	0
14	20	0	0	0	0	0	0	0	0	0	0	0	0
Mean score per animal in 14 days		0			0			0			0		
Mean score per animal daily		0			0			0			0		
